# Delivery of Therapeutic Agents to the Central Nervous System and the Promise of Extracellular Vesicles

**DOI:** 10.3390/pharmaceutics13040492

**Published:** 2021-04-03

**Authors:** Charlotte A. René, Robin J. Parks

**Affiliations:** 1 Regenerative Medicine Program, Ottawa Hospital Research Institute, Ottawa, ON K1H 8L6, Canada; chrene@ohri.ca; 2 Department of Biochemistry, Microbiology, and Immunology, University of Ottawa, Ottawa, ON K1N 65N, Canada; 3 Centre for Neuromuscular Disease, University of Ottawa, Ottawa, ON K1N 6N5, Canada; 4 Department of Medicine, The Ottawa Hospital, Ottawa, ON K1H 8L6, Canada

**Keywords:** central nervous system, blood–brain barrier, therapeutic, extracellular vesicles

## Abstract

The central nervous system (CNS) is surrounded by the blood–brain barrier (BBB), a semipermeable border of endothelial cells that prevents pathogens, solutes and most molecules from non-selectively crossing into the CNS. Thus, the BBB acts to protect the CNS from potentially deleterious insults. Unfortunately, the BBB also frequently presents a significant barrier to therapies, impeding passage of drugs and biologicals to target cells within the CNS. This review provides an overview of different approaches to deliver therapeutics across the BBB, with an emphasis in extracellular vesicles as delivery vehicles to the CNS.

## 1. Introduction

The central nervous system (CNS) is comprised primarily of the brain and spinal cord (in addition to nerves of the olfactory and visual systems), and is tasked with interpreting, coordinating and executing most functions in the body. These functions include movement, sensation, speech, awareness, thought, and memory. Not surprisingly, given such a “central” role in orchestrating life, many of our most debilitating disorders are due to dysfunction of the CNS. Disorders of the CNS include vascular (e.g., stroke), infection (e.g., meningitis), structural (e.g., traumatic brain injury), functional (e.g., epilepsy) and degenerative (e.g., amyotrophic lateral sclerosis (ALS)). New approaches to the treatment of these varied disorders are a goal of many basic and clinical research labs.

The CNS is surrounded by a protective blockade, termed the blood–brain barrier (BBB)—or blood-spinal cord barrier (BSCB) in the case of the spinal cord—a highly selective, semipermeable border of endothelial cells (EC) that prevents pathogens, solutes and most molecules from non-selectively crossing into the CNS. While normally helping to protect the CNS, in the case of disease the BBB and BSCB also function as a “barrier” to therapies, and can prevent effective delivery of therapeutics to target cells or regions within the CNS. In the development of therapeutics for disorders of the CNS, achieving sufficient penetration of the BBB and BSCB is a major hurdle [1]. Since the BSCB shares many properties with the BBB, this review will focus on the BBB, but many of the principles also apply to the BSCB.

## 2. The Blood–Brain Barrier

The BBB is a complex and dynamic interface that regulates the movement of ions, molecules, and cells between the blood and the CNS [2]. Control of CNS homeostasis permits correct neuronal function, and also protects neural tissue from harm due to disease, inflammation, injury, toxins, and pathogens. The primary barrier in the BBB is EC, which line the walls of capillaries that feed into the brain. These EC are connected through tight junctions (TJ) which restrict the passage of substances from the blood more selectively than EC elsewhere in the body. Indeed, EC in the CNS are quite distinct from those of the periphery in several ways. Specifically, EC of the CNS express BBB-specific proteins to control the entry and exit of metabolites across cells (transcellular pathway), possess highly electrical-resistant TJ to limit the flux of molecules between adjacent EC (paracellular pathway), and lack fenestrations (pores which are present in peripheral EC to allow rapid exchange of molecules between blood and tissue), all designed to limit the movement of molecules through the EC barrier [2,3].

**Figure 1:**
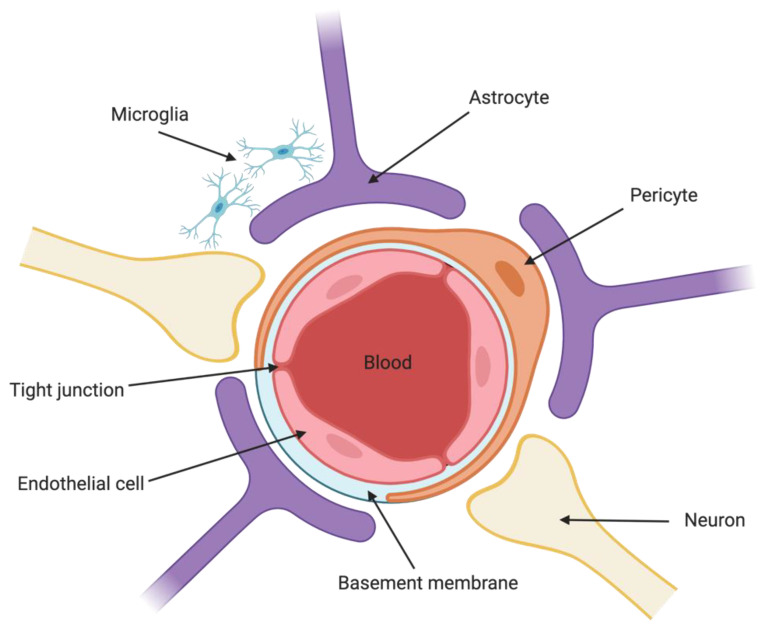
The blood–brain barrier. The BBB is formed by endothelial cells of the capillary wall, the dendrites of neurons and astrocyte end-feet ensheathing the capillary, and pericytes embedded in the capillary basement membrane.

EC are not the only cell type that contribute to BBB selectivity (Figure 1). Pericytes (PC) are embedded in the vascular basement membrane that surrounds EC [4]. CNS microvasculature has an EC:PC ratio of between 1:1 and 1:3, whereas vasculature found in, for example, striated muscle tissue has a ratio of approximately 100:1 [5]. PC are responsible for the regulation of capillary blood flow [6], by modulating capillary diameter through contraction-stimulated intracellular calcium ion (Ca^2+^) concentration [7,8]. BBB PC function in part to maintain proper chemical composition of the surrounding environment, regulate transendothelial fluid transport and paracellular flow between cells, and also protect EC [9]. Astrocytes are major glial cells present at the abluminal side of the BBB, and help to relay signals to regulate blood flow in response to neuronal activity [10]. Neurons are present in close proximity to astrocytic end-feet at the abluminal side of the BBB, and also play a role in regulation of blood flow and microvascular permeability [11].

**Figure 2:**
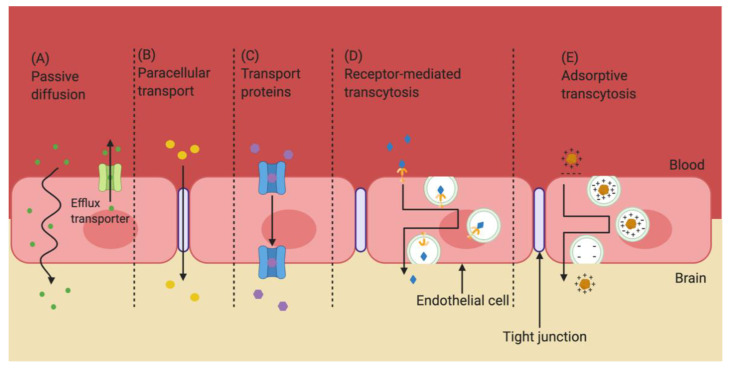
Transport routes across the blood–brain barrier. Molecules can cross the BBB using a variety of mechanisms. Lipophilic molecules of a low molecular weight can diffuse passively through EC (**A**), while hydrophilic molecules of a low molecular weight can pass between EC (**B**). Some solutes use transport proteins to cross the BBB (**C**), while others rely on receptor-mediated transcytosis (**D**) or adsorptive transcytosis (**E**). Adapted from [18], Drug Delivery and Translational Research. 2020.

While the BBB is very selective for what can pass through, the barrier is not completely impenetrable. Some small, lipophilic molecules are able to passively diffuse through EC to cross between the bloodstream and CNS [12,13] (Figure 2A). Water and some small hydrophilic molecules are able to use paracellular transport to cross the BBB [14] (Figure 2B). However, approximately 98% of all small molecules and almost all large molecules (those with a molecular weight greater than 1 kD), are unable to cross the BBB [15]. Most molecules that transit across the BBB do so by interacting with specific receptors and/or transporters that are expressed on EC [16]. For example, small essential hydrophilic compounds, like glucose and amino acids, use transporters that are expressed on both the luminal (blood) and basolateral (brain) side of the EC [16]. Regulation of ion concentrations, including potassium (K^+^), calcium (Ca^2+)^, and magnesium (Mg^2+^), is accomplished through the presence of specific ion channels and transporters (Figure 2C) [17]. Large essential hydrophilic molecules, like hormones and lipoproteins, cross the BBB via endocytosis and transcytosis through use of specific receptors and transporters, respectively, that are highly expressed on the luminal side of EC [3] (Figure 2D,E). In short, the BBB is a very effective barrier that tightly regulates the passage of molecules into the CNS. 

## 3. Methods to Enhance Drug Delivery to the CNS

Invasive approaches are physical techniques that deliver a therapeutic to the CNS by mechanically breaching the BBB [19].

### 3.1. Invasive Approach

#### 3.1.1. Direct Injection

Intra-cerebral injection is an invasive approach that relies on direct injection of a therapeutic into the brain, and subsequent diffusion of the therapeutic from the site of injection into surrounding regions. The injection site must be very precise for this method to be effective, as distribution of therapeutics within the brain by simple diffusion decreases significantly with distance [20]. For example, injection of chemotherapeutic agents into tumor resection cavities has been used in the treatment of brain tumors [21,22,23,24]. Direct injection of recombinant adeno-associated virus (AAV) expressing various factors has been studied for treatment of CNS disorders, such as Spinal Muscular Atrophy (SMA) [25], Niemann-Pick type C1 disease (NP-C) [26], Leigh Syndrome (LS) [27] in mouse models, as well as Parkinson’s disease (PD) in monkeys [28] and patients [29], and Alzheimer’s disease (AD) in mouse models [30,31]. Delivery to the cerebral ventricle of a self-complimentary AAV serotype 9 (AAV9) encoding a codon-optimized human survival motor neuron 1 gene (*SMN1*) led to a dose-dependent rescue of lifespan and growth [25]. Similarly, direct injection of an AAV9 encoding a human intracellular cholesterol transporter 1 gene (*NCP1*) improved lifespan, locomotor function, and disease pathology in a mouse model of NP-C [26]. The relative efficacy of direct injection into cerebral ventricles versus systemic intravenous (IV) delivery was compared in a mouse model of LS, using an therapeutic AAV2/9 encoding the human ubiquinone oxidoreductase iron-sulfur protein 4 (*NDUFS4*) gene [27]. This study concluded that systemic IV administration was able to correct disease pathology only in peripheral organs, whereas direct injection was able to partially improve disease pathology only in the brain, and neither treatment was able to improve the lifespan of the mice. [27]. However, treatment with both methods of administration was able to significantly improve lifespan and disease pathology. 

#### 3.1.2. Intracerebroventricular Infusion

Intracerebroventricular (ICV) infusions involve prolonged administration of a therapeutic substance directly into the CSF contained in the cerebral ventricle. Administration can be achieved through use of an implanted osmotic pump or IV catheter. ICV infusions can occur over the course of several days or weeks, and delivery to the brain parenchyma is accomplished by simple diffusion [32,33,34]. 

ICV infusion of dopamine or dopamine agonists via a catheter implanted in the cerebral ventricle in a rat model of PD were found to restore dopamine levels in the CNS [35]. Similarly, ICV infusion of these compounds restored normal behavior patterns in monkeys that had been rendered akinetic through treatment with 1-methyl-4-phenyl-1,2,3,6-tetrahydropyridine (MPTP) [35]. However, the ICV infusion was complicated due to intolerance to the pump, or frequent disconnection of the pump from the catheter [35]. Infusion of recombinant human platelet derived growth factor-BB (rhPDGF-BB) over the course of two weeks in rats with 6-hydroxydopamine-induced nigrostriatal lesions resulted in the restoration of striatal dopamine transporter binding sites and expression of nigral tyrosine hydrolase (TH), and normalized amphetamine-induced rotational behavior [36]. ICV infusion of rhPDGF-BB was used safely in a human clinical trial for treatment of PD, reducing Parkinsonian symptoms and may have increased the integrity and function of dopaminergic neurons [37]. ICV infusion of anti-β-secretase (BACE1) in non-human primates resulted in a sustained reduction of 70% of amyloid-β peptides in the CSF, suggesting a possible therapeutic avenue for neurodegenerative disorders such as AD and PD [38]. Unfortunately, ICV infusions are not an efficient method of achieving brain-wide drug delivery, but may be applicable to cases in which the target region is in close proximity to the cerebral ventricles [20].

#### 3.1.3. Convection Enhanced Delivery

Convection enhanced delivery (CED) seeks to increase the overall area of the brain that receives exposure to a therapeutic. CED involves stereotactically guided insertion of a cannula into the brain parenchyma, followed by active pumping of a drug through the cannula over several hours or days [39]. CED is thus powered by the pressure gradient created through use of the pump, which allows for greater dispersion of a therapeutic relative to standard diffusion [40,41,42]. CED was used in a phase 1 clinical trial to investigate the efficacy of [^124^I]-labelled 8H9 murine monoclonal antibody (MAb) for treatment of diffuse intrinsic pontine glioma in children [43]. 8H9 binds B7-H3, a surface antigen and immune modulator of natural killer and T cells, and is overexpressed in most high-grade gliomas [44]. CED delivery of [^124^I]-labelled 8H9 was deemed safe, with no treatment-related deaths or grade 4 adverse events, and patient survival was extended, although this latter point may have been confounded by potential patient selection bias [43]. Limitations of CED include that optimal therapeutic delivery is contingent on proper placement of the catheter, and that some areas of the brain are difficult to saturate fully with a therapeutic by CED, particularly areas surrounding a cavity [45]. Some studies comparing CED to direct injection have noted greater distribution and efficacy with bolus injection [24,46].

#### 3.1.4. Barrier Disruption

The BBB can also be physically breached by disrupting the connections between EC, causing leakage between cells [17]. One method to compromise the integrity of the BBB is through osmotic disruption, in which EC shrink due to application of osmotic shock, thereby increasing the gap between cells [16]. Pretreatment by carotid artery injection of 25% mannitol, to induce osmotic shock and disruption of the BBB, led to significantly enhanced uptake of hydrophobically modified siRNA (hsiRNA) and subsequent target-gene silencing in rat brain [47]. 

Magnetic resonance imaging (MRI)-guided focused ultrasound can also be used to disrupt the BBB [16]. In animal models of cancer, disruption of the BBB using MRI-guided focused ultrasound enhanced delivery of chemotherapeutic agents such as bevacizumab, temozolomide (TMZ), and doxorubicin (DOX), slowing tumor progression and improving survival [48]. Focused ultrasound combined with viral microbubbles carrying brain-derived neurotrophic factor (BDNF) have been explored as a potential therapeutic in a rat model of AD [49]. However, this approach requires that the animal/patient is anesthetized and can be costly, and neurons can suffer permanent damage due to influx of blood component into the brain [50,51]. 

### 3.2. Lipophilic Approach

Lipophilic approaches to enhance drug delivery to the CNS involve modification of drugs at the molecular level to increase passive movement across the BBB [19]. For example, reducing the relative number of polar groups in a drug increases its transport across the BBB [52]. Covalent addition of a 1-methyl-1,4-dihydronicotinate moiety to the hydroxymethyl group of ganciclovir enhanced delivery to the brain for treatment of cytomegalovirus-induced encephalitis [53]. Ganciclovir is a prodrug and must be converted to an active form for efficacy. Direct-acting compounds can also be modified to enhance passage across the BBB, such as use of pyrrolopyrimidines to modify antioxidants to increase their ability to reach target cells in the CNS [54]. However, although these molecular changes can enhance the ability of compounds to cross the BBB, the modifications may also alter the functionality of the compound, thereby reducing effectiveness [19].

### 3.3. Physiological Approach

#### 3.3.1. Receptor-Mediated Transcytosis

**Figure 3:**
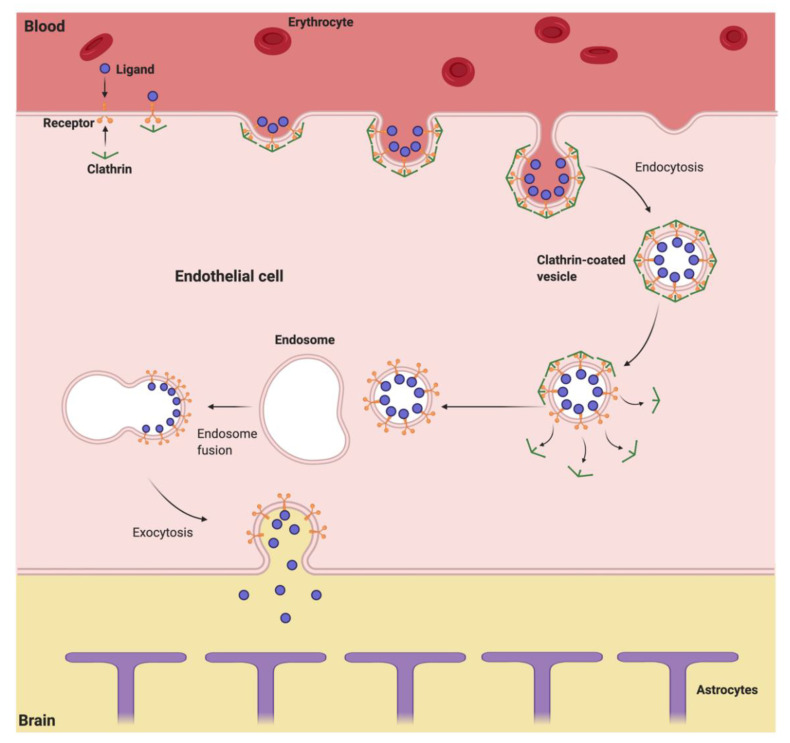
Receptor-mediated transcytosis. The ligand engages with the receptor at the luminal side of the BBB, followed by receptor-mediated endocytosis of the receptor-ligand complex into the EC, forming a clathrin-coated vesicle. The vesicle fuses with the endosome, after which the vesicle undergoes exocytosis, and the biomolecule is released on the abluminal side of the BBB.

Large molecules that require access to the brain typically utilize receptor-mediated transcytosis (Figure 3), a multi-step process involving specific receptors expressed on the EC, such as transferrin receptors [55,56], insulin receptors [57,58], and low-density lipoprotein (LDL) receptor related proteins (LRP) [59]. The process of receptor-mediated transcytosis, also called clathrin-mediated endocytosis, begins with engagement of ligand with the receptor located on the luminal side of the BBB, followed by receptor-mediated endocytosis of the ligand-receptor complex into the EC, forming a clathrin-coated vesicle within the cell. This vesicle then fuses with the early endosome in the cytoplasm of the EC. Acidification of the endosome results in dissociation of the biomolecule from the receptor [60]. The acidified endosome then transits to the abluminal side of the EC, and fusion of the vesicle with the cell membrane releases the biomolecule into the CNS [20,61].

Transferrin receptors are responsible for facilitating transcytosis of transferrin-coupled iron to the brain parenchyma [20]. Drugs can be targeted to the transferrin receptors through conjugation to transferrin or antibodies (Ab) specific to the transferrin receptor in human tissue culture [62,63], rats [64], and primates [63]. Several studies have used this approach to delivery therapeutics to the CNS, including vasoactive intestinal peptide (VIP) [65], epidermal growth factor (EGF) [66], amyloid β1–40 peptide (Aβ1-40) [67], and β-galactosidase [68]. Recently, this approach has been used in clinical trials to lower levels of heparin sulfate in CSF, showing promise as a treatment for Hunter syndrome [69]. Indeed, several companies have now advanced transferrin-conjugated therapeutics into clinical trial for treatment of a variety of CNS disorders [69]. A similar approach has used Ab to the insulin receptor in primate studies to enhance CNS delivery of BDNF, fibroblast growth factor-2 (FGF-2), siRNA, and neurotrophin [70]. 

The LDL receptor related proteins 1 and 2 (LRP-1 and -2) have also been used to transport target drugs to the CNS. LRP is a multi-ligand, transmembrane protein that is expressed on many cells of the CNS, including EC [59], neurons [71,72], and astrocytes [72]. LRP naturally transports apolipoprotein E and B into the CNS, and it is hypothesized that some therapeutic nanoparticles may naturally preferentially bind these apolipoproteins, which facilitates transcytosis via LRP [73]. Alternatively, fusion proteins can be created that specifically bind LRP, thus facilitating uptake. For example, fusion of the lysosomal enzyme glucocerebrosidase to the low-density lipoprotein receptor-binding domain of apolipoprotein B resulted in effective delivery of the protein to the CNS by LPR-mediated transcytosis in mice [74]. 

Rabies virus naturally infects neurons [75], and a peptide derived from the rabies virus glycoprotein (RVG), which binds the nicotinic acetylcholine receptor, was shown to facilitate delivery of therapeutic siRNA to the brain following systemic delivery [76]. In this approach, severe combined immunodeficient (SCID) mice were infected with Japanese Encephalitis Virus (JEV) and subsequently treated with IV injection of JEV-directed siRNA (siFvE^J^) complexed with the RVG peptide. Treated mice showed 80% survival, compared to 100% lethality in untreated mice, suggesting that siFvE^J^/RVG-peptide may be an effective therapeutic for treatment of JEV-induced encephalitis.

Innovative formulation technologies, such as nanoparticles that are engineered to bind receptors on the BBB, show promise as an effective platform for delivery of therapeutics to the CNS [77]. Nanoparticles bonded to transferrin or MAb against transferrin receptors were used to successfully deliver loperamide, a model drug, into the brain [78]. Receptor-mediated transcytosis can also be used to transport plasmid DNA across the BBB [79]. For example, liposome-encapsulated DNA can be coated with polyethylene glycol (PEG), and then conjugated with MAb specific for an appropriate receptor, creating pegylated immunoliposomes (PIL) [80]. IV administration of a PIL-encapsulated plasmid encoding TH under regulation by the glial fibrillary acidic protein (GFAP) promoter resulted in an 82% reduction in apomorphine-induced rotation in a rat model of PD [81]. PIL conjugated to a murine MAb that binds the human insulin receptor were used to deliver a human EGFR antisense gene to treat a U87 human glioma cell xenograft brain tumor mouse model of cancer, and resulted in a 70–80% inhibition in cancer cell growth [82].

Although promising, receptor-mediated transcytosis does have potential drawbacks. Some studies have shown that nanoparticles have a tendency to accumulate in brain capillary EC, which reduces their effective delivery to the target tissue in rats [83,84]. As there are a finite number of receptors on both the luminal and abluminal sides of the BBB, receptor-mediated transcytosis is a saturable process [85], which may limit the concentration of the delivered drug that can be achieve in the brain parenchyma [86,87]. Finally, there is the concern of potential immunogenicity, or even toxicity, due to use of non-human Ab [88].

#### 3.3.2. Transporter-Mediated Processes

Drugs that closely resemble a molecule that naturally transits the BBB by a transporter are able to take advantage of this transporter system [20]. For example, L-Dopa naturally has high affinity for the amino acid transporter system L [89], and has been used to deliver L-Dopa to treat PD [90]. Alternatively, a drug can be conjugated to a molecule naturally transported through this system, thus allowing co-transport of the drug. A plasmid encoding human tumor necrosis factor-related apoptosis-inducing ligand (TRAIL) was complexed with DOX, and subsequently conjugated to a choline derivate, for transport by the choline transporter [88]. The resulting complex showed greater uptake in human neuronal glioblastoma-astrocytoma U87 MG cells and enhanced ability to induce apoptosis in vivo and in vitro compared to each component alone. To use a transporter protein to enhance drug delivery through the BBB, the structural binding requirements of the transporter and possible deleterious effects due to modification of the therapeutic compound must be considered [16]. 

Once in the CNS, inhibition of efflux transports can be used to maintain levels of the drug. The antibiotic cefadroxil is a substrate for multiple transporters present at the BBB, such as organic anion transporters (OATs) [91,92], organic anion transporting polypeptides (OATPs) [93], multidrug resistance-associated proteins (MRPs) [94,95], and peptide transporter 2 (PEPT2) [96], some of which can efflux the drug. Inhibition of OATs, OATPs, and MRPs with probenecid significantly increased the concentration of cefadroxil, a model drug, in brain extracellular fluid due to inhibition of cefadroxil efflux at the BBB [97]. Brain slice experiments indicated that PEPT2 is involved in uptake of cefadoxil, however inhibiting PEPT2 with Ala-Ala did not increase brain ECF cefadroxil levels.

#### 3.3.3. Adsorptive Mediated Endocytosis

Adsorptive mediated endocytosis is triggered by the uptake of cationic molecules into endocytic vesicles on the luminal surface of the BBB, and subsequent fusion of the vesicles at the abluminal membrane, resulting in release of the endocytic contents into the brain parenchyma. Compounds such as avidin, histone, and protamine rely on adsorptive mediated endocytosis to cross the BBB [98,99]. As adsorptive mediated endocytosis requires a substance to be cationic to facilitate binding of the molecule to the negatively charged cell surface, cationizing can allow other molecules to utilize this transport process to enter the CNS. Chemical cationization of a protein is possible through the amidation of carboxylic acid groups on the protein [100]. Covalent addition of hexamethylenediamine to anti-tetanus Ab fragments cationized the fragments and allowed entry of the molecule into the CNS [101]. This cationization did not alter the affinity of the Ab fragment for the tetanus toxin in vitro, and showed promise as a therapeutic strategy for treatment of tetanus. Similarly, putrescene-modified catalase was shown to increase the ability of catalase to cross the BBB in mouse models of ALS [102,103]. Limitations of chemical cationization include potential deleterious effects of the modification on protein function, and formation of immune responses against the modified protein [104]. There is also the concern of inducing immune complex-mediated glomerulonephritis, as cationic antigens and immune complexes containing cationic Ab can deposit in the glomeruli [105].

An alternative to chemical cationization is through the use of cell-penetrating peptides (CPP). CPP are small peptides with positive charge that are derived from proteins that have a natural ability to bind to and cross the cell membrane, such as the human immunodeficiency virus (HIV) transactivator of transcription (Tat) protein, first described in a human leiomyosarcoma cell line, SK-LSM [106]. CPP can be attached to biomolecules or to the surface of nanoparticles, and mediate delivery to the CNS. This approach was used to deliver ciprofloxacin, a model antibiotic, across the BBB as a potential treatment for infections of the CNS [107]. In another study, the surface of chitosan (CS)-coated nanostructured lipid carriers (NLC) were modified with Tat (CS-NLC-Tat), and used to deliver glial cell-derived neurotrophic factor (GDNF) (CS-NLC-Tat-GDNF) to a mouse model of PD [108]. Treated mice exhibited significant motor recovery, as well as increased numbers of TH+ fibers in the striatum and TH+ neurons in the substantia nigra, indicating that intranasal delivery of CS-NLC-Tat-GDNF may be a promising therapy for treatment of PD [108]. However, use of CPP have raised some concerns due to toxicity, as Tat can induce toxicity in cells in culture [109] and in rat neurons [110] and can also induce apoptosis of EC [111].

## 4. Extracellular Vesicles as Delivery Vehicles to the CNS

Many groups have shown that extracellular vesicles (EV) have a natural ability to transit the BBB and deliver biomolecules to the CNS. EV are tiny, membrane bound particles released from all cell types and found in all bodily fluids [112]. Although originally thought to represent cellular waste, EV actually perform an array of physiological functions in the body [113]. EV are naturally involved in intercellular communication, transferring proteins, lipids, and nucleic acids to recipient cells which may be located some distance from the site of EV origin [114]. Specific function and physical composition of the vesicle depends on the type of cell from which the EV originates [112].

### 4.1. EV Biogenesis

**Figure 4:**
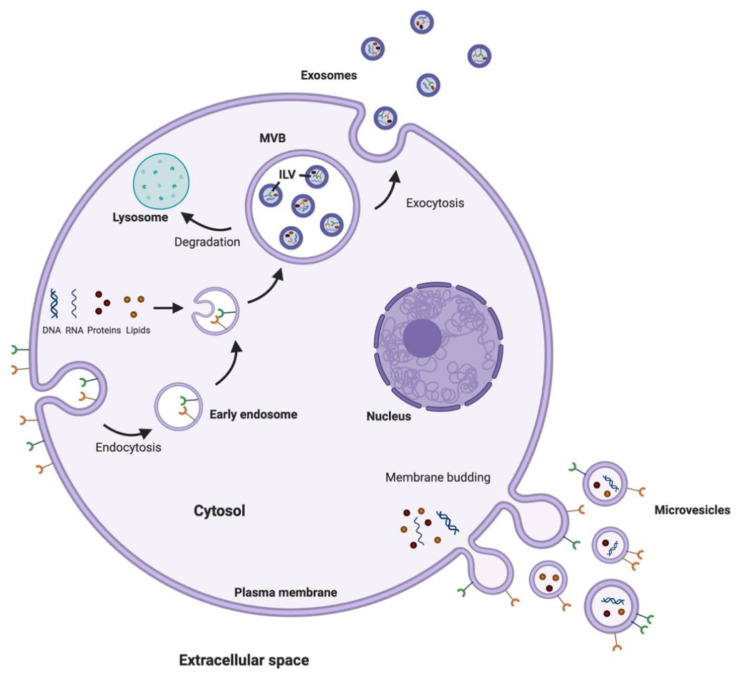
Biogenesis of microvesicles and exosomes. Microvesicles are generated by budding of the plasma membrane. Exosomes are formed through the endosomal network and released into the extracellular space after the MVB containing intraluminal vesicles (ILV) fuses with the plasma membrane.

There are three main subtypes of EV that are differentiated based upon their biogenesis, release pathways, size, content, and function; exosomes, microvesicles (MV), and apoptotic bodies (Figure 4). Exosomes are the smallest, with vesicles having a diameter of between 40–100 nm [115]. Exosomes are formed within the endosomal network in multivesicular bodies (MVB) and subsequently released into the extracellular environment after fusion of the MVB with the plasma membrane [116]. MV are slightly larger, generally considered to be 100–1000 nm in diameter, and are generated by budding at the plasma membrane [112,115]. The final class of EV are apoptotic bodies, which form through blebbing from the plasma membrane of dying cells. Apoptotic bodies are the largest classification of EV, with a diameter over 1000 nm [115]. 

Exosomes and MV are continually released under normal conditions but their release is upregulated under times of stress [112]. This enhanced release of EV may function as a method of eliminating waste and potentially toxic cellular contents from the cell, as well as a way to communicate intracellular stress to nearby or distant cells [115]. Obviously, apoptotic bodies are only released as a result of severe and terminal cellular insult. 

Knowing which class of EV is used in a particular study can be somewhat challenging. Current methods of isolation are not capable of fully separating the various classes of EV, only enriching for certain size populations (e.g., “large” versus “small” EV). Moreover, the lack of standard methods for isolating EV makes it difficult to know what class of EV was actually used or to compare between studies. Finally, the term “exosome” is frequently used synonymously with EV, regardless of particle size. The International Society of Extracellular Vesicles has attempted to clarify some of these points with strong guidelines for EV terminology, isolation and characterization [117]. In the studies described below, we will utilize the terminology used in the cited work.

### 4.2. EV Uptake and Mechanism of Transit Across the BBB

As mentioned, one of the functions of EV appears to be to transmit signals between cells located both near and far from the cell of origin. Once bound to the plasma membrane of the target cell, EV can dissociate, remain stably associated, fuse with the plasma membrane, or be internalized by the cell. The mechanism of uptake of EV by a target cell is dependent on the recipient cell type [112], and can include endocytosis, clathrin-dependent endocytosis, caveolin-dependent endocytosis, phagocytosis, and macropinocytosis [118].

The mechanism that EV use to cross the BBB has yet to be fully elucidated. In an in vitro model of the BBB, purified exosomes appeared to be internalized by EC via endocytosis, either by a clathrin- or caveolin-dependent pathway, and subsequently trafficked via endocytic mechanisms [119]. Interestingly, treatment of this model with the pro-inflammatory cytokine TNF-α, to mimic stroke-like conditions, increased the permeability of the EC monolayer, allowing passage of the EV by a transcellular route [119]. In the absence of TNF-α, EV were not found in the abluminal side of the EC. Thus, the route or efficiency of EV passage through the BBB may be strongly influenced by the inflammatory state of the host.

Transit of EV across the BBB has also been examined using tumor-derived EV [120,121]. Administration of purified MDA-MB-231 breast cancer cell-derived EV to an in vitro model of the BBB, or following cardiac injection of zebrafish in vivo, showed that EV cross the brain endothelium via transcytosis [120]. In this system, transcytosis appeared to involve caveolin-independent, clathrin-dependent endocytosis. In an in vitro study examining the mechanism of binding and internalization of exosomes derived from a brain-metastatic melanoma cell line (SK-Mel-28) in human BBB-derived EC (hCMEC/D3 cells), CD46 was determined to be a major receptor for exosome uptake in this cell type [121]. 

### 4.3. Therapeutic Applications

EV have a number of advantages as biotherapeutics [122]. Cells release EV continuously, and thus large quantities of therapeutic EV can be produced relatively easily. EV are also stable ex vivo, allowing for easy storage of therapeutic stocks. EV are resistant to breakdown in the bloodstream, and the lipid bilayer thus protects cargo from degradation. The hydrophilic shell of EV contain anti-phagocytosis surface markers, such as CD47, that enable the vesicles to evade phagocytosis by macrophages and monocytes, limiting their clearance by the recticulo-endothelial system [123,124]. Therapeutic EV induce minimal immunogenicity and toxicity in vitro and in vivo [125,126]. Finally, as mentioned, EV that are in circulation are capable of crossing the BBB, and are able to move both into and out of the CNS [122]. 

EV can be loaded with therapeutic cargo using either exogenous or endogenous loading. In exogenous loading, EV are first isolated and purified, and then compounds are incorporated through methods such as co-incubation, electroporation, permeabilization, freeze–thaw cycles, extrusion, or sonication [127,128,129]. In endogenous loading, the cells that will ultimately release the EV are manipulated, for example through engineering to overexpress a particular therapeutic protein, so that the protein is incorporated in the released EV [130,131]. EV can also be modified to enhance uptake by cells of the CNS. Expression of a fusion protein comprised of Lamp2b (normally found in the membrane of exosomes) with an RVG peptide allows presentation of the peptide on the surface of the exosomes, resulting in enhanced targeting and uptake by neurons [132].

#### 4.3.1. EV as a Vehicle for Delivery of Small Molecules

Curcumin (Cur) is well known for its anti-inflammatory, anti-cancer, anti-microbial, anti-diabetic, and neuroprotective properties [133,134,135,136,137,138]. Unfortunately, Cur shows poor stability and bioavailability, which has limited its therapeutic use. In a mouse model of LPS-induced encephalitis, Cur loaded into exosomes showed increased stability and bioavailability compared to Cur alone, thus providing a greater protective effect in vivo [128]. To prepare the therapeutic EV, Cur was mixed with murine lymphoma cell (EL4)-derived exosomes and subjected to a sucrose concentration gradient to load Cur into the exosomes. Use of a concentration gradient alters the osmolarity of the exosomes, allowing a compound of interest, such as Cur, to flow into the exosomes. When administered intranasally, Cur-loaded exosomes induced microglial apoptosis, which in turn led to a decrease in inflammation in the brain [128]. Intranasal administration of exosomes loaded with either Cur or JSI124, an inhibitor of Stat3 signaling, protected mice against LPS-induced brain inflammation, and showed beneficial effects in an model of autoimmune encephalomyelitis (EAE) and a GL26 tumor model [133].

Drug-loaded exosomes have also been used for a combined imaging and therapeutic application. In this study, exosomes were loaded with Cur and superparamagnetic iron oxide nanoparticles (SPION) by electroporation. SPION can function as a negative contrast agent for MRI, as well as provide a therapeutic effect through magnetic fluid hyperthermia (MFH) [139,140]. The resulting Cur/SPION exosomes were then conjugated with a neuropilin-1-targeting peptide (RGE) (RGE-Exo-SPION/Cur), to facilitate targeting to glioma cells [139]. Following tail vein delivery in a mouse model of glioma, the RGE-Exo-SPION/Cur allowed for targeted imaging of glioma tumors, which may aid in early detection of the disease. Importantly, administration of RGE-Exo-SPION/Cur significantly inhibited tumor growth, and appeared to act synergistically compared to RGE-Exo-SPION or RGE-Exo-Cur alone [139]. 

IV delivery of mesenchymal stromal cell (MSC)-derived exosomes conjugated to cyclo(Arg-Glys-Asp-D-Tyr-Lys) (c(RGDyK)), a peptide that has high affinity for α_v_β_3_, was shown to target the lesion region in an ischemic brain [141]. These exosomes were loaded with Cur and administered to a transient middle cerebral artery occlusion (MCAO) mouse model of ischemic stroke. Administration of these therapeutic exosomes significantly suppressed the inflammatory response and reduced cellular apoptosis in the lesion region. Results of this study suggest that targeted exosomes loaded with Cur may serve as an effective treatment following ischemic stroke.

Exosomes have also been explored as a delivery vehicle for anti-cancer drugs to the brain. Exosomes isolated from brain endothelial bEND.2 cells were loaded with DOX by simple coincubation [142]. Zebrafish embryos that had been injected in the brain ventricle with U87 MG tumor cells were treated with the therapeutic EV by cardiac vein injection. Embryos treated with these therapeutic EV exhibited reduced tumor cell growth compared to embryos treated with the drug alone or buffer control. Exosome-delivered DOX was also found to significantly suppress vascular endothelial growth factor (VEGF) mRNA levels within the tumor, a surrogate marker of cancer growth [142]. Similarly, human endometrial stem cell-derived exosomes loaded with atorvastatin were effective in inducing apoptosis in U87 cells and also in inhibiting tumor growth in a 3D glioblastoma model [143]. 

#### 4.3.2. EV as a Vehicle for Delivery of Nucleic Acids

EV can also be used to deliver therapeutic nucleic acids to the CNS. Coincubation of hsiRNAs targeted to Huntingtin mRNA (hsiRNA^HTT^) with purified exosomes resulted in efficient loading [144]. hsiRNA^HTT^-loaded exosomes were administered by ICV infusion over the course of 7 days, and were effective in reducing Huntingtin mRNA levels by up to 35% in the mouse brain. Cells engineered to overexpress a specific miRNA can also be used as a source of therapeutic EV. miR-133b is known to promote neurovascular plasticity [145,146], MSC transduced with a lentiviral vector to overexpress miR-133b released exosomes with increased levels of the miRNA [147]. Intra-arterially delivery of miR-133b-containing exosomes to a middle cerebral artery occlusion (MCAO) model of stroke in rats significantly improved functional recovery and increased neural plasticity. Similarly, exosomes isolated from EPC engineered to express elevated levels of miR-137 were able to provide a neuroprotective effect against apoptosis and mitochondrial dysfunction in SH-SY5Y human neuroblastoma cells [148]. Finally, stimulation of dendritic cells (DC) with IFN-γ causes release of EV enriched with miRNA-219 which, when applied intranasally, reduced oxidative stress and regulated oligodendrocyte remyelination in vivo [149], suggesting a novel therapeutic for multiple sclerosis. 

As mentioned, presentation of the RVG peptide on the surface of EV can enhance homing of systemically delivered EV to the brain [132]. Electroporation-mediated loading of these CNS-targeted exosomes with siRNA to BACE1, a therapeutic target in AD, resulted in knockdown of BACE1 mRNA (60%) and protein (62%) in the brain of mice [132]. Similarly, systemic delivery of RVG-targeted exosomes loaded with siRNA to α-synuclein to a mouse model of PD resulted in a decrease in α-synuclein by 84%, and decreased α-syn-mRNA by 49% in the midbrain, 56% in the striatum, and 50% in the cortex [150].

Circular RNA (circRNA) have covalently connected 5′ and 3′ terminal ends, creating a closed long non-coding RNA (lncRNA) [151]. CircSHOC2, a circRNA transcribed from the SHOC2 gene, delivered in exosomes suppressed apoptosis and ameliorated neuronal damage in an ischemic stroke model by acting through the miR-7670-3p/SIRT1 pathway to regulate autophagy [152]. In this study, ischemic-preconditioned astrocyte-derived exosomes (IPAS-Exo) were used, as these exosomes were found to have significantly increased levels of CircSHOC2 caused by ischemic treatment. 

#### 4.3.3. EV as a Vehicle for Delivery of Therapeutic Proteins

EV can also be used to deliver therapeutic proteins. Delivery of exosomes loaded with catalase promoted neuroprotective effects following intra-nasal delivery in a mouse model of PD [129]. Exosomes were loaded with catalase by several different methods, including sonication, freeze–thaw, extrusion, permeabilization with saponin, or co-incubation at room temperature. Exosomes loaded through sonication, extrusion, or saponin permeabilization were found to have the most significant loading and release of catalase. Administration of the catalase-loaded exosomes provided a significant neuroprotective effect in a mouse model of PD, with exosomes loaded utilizing saponin permeabilization providing the greatest effect. More recently, EV were used to deliver Neprilysin (NEP), an enzyme involved in the clearance of aggregated β-amyloid sheets in the brain [153,154]. Bone marrow-derived MSCs were used as a source of the EV, and NEP was loaded into the EV by freeze–thaw cycles, which interrupts the integrity of the EV membrane allowing internalization of NEP. Intranasal delivery of NEP-EV in a rat model of AD resulted in an increase in expression of B cell leukemia/lymphoma 2 (BCL2), an anti-apoptotic factor, and also a decrease in expression of both IL-1β, an inflammation factor, and BCL2-associated X protein (BAX), a pro-apoptotic factor, in the rat brain, overall improving brain-related behavioural function [153]. 

More recently, ARMMs, a new class of EV [155], have been explored as a potential therapeutic vehicle. A HEK293T-based cell line was generated that stably expressed a fusion of the tumor-suppressor p53 protein to ARRDC1 (a protein naturally loaded into ARMMs), resulting in enhanced loading of the p53 fusion protein [155,156]. IV delivery of ARRCD1-p53 ARMMS resulted in protein delivery to multiple tissues, and induced significant DNA damage-dependent apoptosis in the thymus and spleen in p53-null mice. This approach may represent a novel therapeutic against many cancers in which the function of p53 is compromised [156].

## 5. Conclusions

In this review, current approaches for delivering therapeutics across the BBB and into the CNS have been discussed. Physiological, lipophilic, and invasive methods can effectively deliver therapeutics across the BBB, but each is associated with technical problems or safety concerns. EV have gained attention in the last several years as possible delivery vehicles for therapeutic compounds, including nucleic acids, proteins, and small molecule drugs. EV have shown great promise for delivery to the CNS in varied models of disease, and it is hoped that continued development and refinement in EV technology will further improve their utility for treating diseases of the CNS.

## Data Availability

Not applicable.
